# Evidence of a multiple insecticide resistance in the malaria vector *Anopheles funestus* in South West Nigeria

**DOI:** 10.1186/s12936-016-1615-9

**Published:** 2016-11-22

**Authors:** Rousseau J. Djouaka, Seun M. Atoyebi, Genevieve M. Tchigossou, Jacob M. Riveron, Helen Irving, Romaric Akoton, Michael O. Kusimo, Adekunle A. Bakare, Charles S. Wondji

**Affiliations:** 1 International Institute of Tropical Agriculture, 08 BP 0932, Cotonou, Benin; 2 Cell Biology and Genetics Unit, Department of Zoology, University of Ibadan, Ibadan, Oyo State Nigeria; 3 Liverpool School of Tropical Medicine, Pembroke Place, Liverpool, L3 5QA UK; 4 University of Abomey Calavi, BP 526, Cotonou, Benin

**Keywords:** Malaria control, *Anopheles funestus*, Insecticide resistance, Resistance mechanisms, Nigeria

## Abstract

**Background:**

Knowing the extent and spread of insecticide resistance in malaria vectors is vital to successfully manage insecticide resistance in Africa. This information in the main malaria vector, *Anopheles funestus* sensu stricto, is completely lacking in the most populous country in Africa, Nigeria. This study reports the insecticide susceptibility status and the molecular basis of resistance of *An. funestus* as well as its involvement in malaria transmission in Akaka-Remo, a farm settlement village in southwest Nigeria.

**Results:**

*Plasmodium* infection analysis using TaqMan protocol coupled with a nested PCR revealed an infection rate of 8% in *An. funestus s.s*. from Akaka-Remo. WHO susceptibility tests showed this species has developed multiple resistance to insecticides in the study area. *Anopheles funestus s.s.* population in Akaka-Remo is highly resistant to organochlorines: dieldrin (8%) and DDT (10%). Resistance was also observed against pyrethroids: permethrin (68%) and deltamethrin (87%), and the carbamate bendiocarb (84%). Mortality rate with DDT slightly increased (from 10 to 30%, n = 45) after PBO pre-exposure indicating that cytochrome P450s play little role in DDT resistance while high mortalities were recorded after PBO pre-exposure with permethrin (from 68 to 100%, n = 70) and dieldrin (from 8 to 100%, n = 48) suggesting the implication of P450s in the observed permethrin and dieldrin resistance. High frequencies of resistant allele, 119F in F_0_ (77%) and F_1_ (80% in resistant and 72% in susceptible) populations with an odd ratio of 1.56 (P = 0.1859) show that L119F-GSTe2 mutation is almost fixed in the population. Genotyping of the A296S-RDL mutation in both F_0_ and F_1_ samples shows an association with dieldrin resistance with an odd ratio of 81 (P < 0.0001) (allelic frequency (R) = 76% for F_0_; for F_1_, 90 and 10% were observed in resistant and susceptible populations, respectively) as this mutation is not yet fixed in the population.

**Conclusion:**

The study reports multiple insecticide resistance in *An. funestus* from Akaka Remo. It is, therefore, necessary to pay more attention to this major malaria vector for effective malaria control in Nigeria.

## Background

Malaria remains the most severe infectious disease and a major public health challenge in Nigeria [1, 2]. It is the main cause of morbidity and mortality in this most populous Africa country, with 97% of the national population at risk: it is responsible for an estimated 300,000 deaths annually in Nigeria; and it contributes to an estimated 11% maternal mortality as well as 25% of infant mortality [1, 3]. Malaria transmission in Nigeria has been attributed mainly to *Anopheles gambiae* sensu stricto (*s.s.*) and *Anopheles funestus s.s.* [4–6] with consistent *Plasmodium* infection rates of 1.0–2.7% (*An. funestus*) and 3.0–8.1% (*An. gambiae*) previously reported in case studies in Ogun, Oyo and Lagos states [4, 5]. Although, there was also a high sporozoite infection rate of 25% reported in Lagos state [7]. In Nigeria, malaria control relies hugely on the use of indoor residual spraying (IRS) and insecticide-treated nets (ITNs) [2, 3]. However, resistance against the main insecticides used in public health (pyrethroids, carbamates and organochlorines) in malaria vectors is threatening the effectiveness of these control tools. *Anopheles gambiae s.s* resistance to insecticides, notably against pyrethroids [8], DDT [9, 10] and bendiocarb [11], has been documented in Nigeria, however, little is known so far concerning the insecticide susceptibility of the other major malaria vector *An. funestus s.s.* in the country. Pyrethroid insecticide is the class of insecticide mainly used in Nigeria for both ITNs and IRS [2]. Two types of pyrethroids are mainly used in Nigeria for insecticide nets treatment: permethrin (Type 1) and deltamethrin (Type 2). In recent years, *An. funestus s.s.* populations have increasingly been reported to be resistant to these insecticides in other African countries, such as Uganda in East Africa [12]; Mozambique, Zambia, Zimbabwe and Malawi in Southern Africa [13–19], Cameroon in Central Africa [20, 21] and some West African countries, including Benin [22], Ghana [23] and Burkina-Faso [24]. Resistance patterns against these insecticides vary significantly across Africa. For example, *An. funestus* was resistant to pyrethroids and carbamate but fully susceptible to DDT and dieldrin in southern Africa [20, 25]. However, a recent study in Malawi showed that this mosquito species has now began to develop resistance against organochlorines (dieldrin and DDT) [17]. *Anopheles funestus* is resistant to pyrethroids and DDT, but remains susceptible to carbamate in Uganda and western Kenya [12]. High resistance profiles were recorded with dieldrin in Cameroon [20]. In the neighbouring country of Benin, resistance firstly reported in 2011 [22] from the coast (Pahou) and was recently shown to have extended to the inland as the Kpome population was shown to be resistant to all insecticide classes apart from organophosphates [26]. It remains to be established whether these resistances are also present in Nigeria and if yes information on the resistance pattern will be useful for the malaria control programs especially on the suitable insecticides to use for the control of this species.

Metabolic resistance mechanisms have so far been implicated in insecticide-resistant *An. funestus* across Africa [12, 22, 27] with cytochrome P450 genes conferring pyrethroid resistance and also cross-resistance to carbamates in southern African [28] as previously reported also for *An. gambiae* [29]. DDT resistance mechanisms in *An. funestus* on the other hand have been associated with an up-regulation of glutathione S-transferases notably *GSTe2* coupled with a point mutation L119F [27]. No L1014F-kdr mutation has been implicated in pyrethroids and DDT resistance [12, 22], and no association exists between G119S and F455W mutations of the *Ace*-*1* gene and carbamate resistance in this mosquito species [12, 22, 25]. However, the recent discovery of a new *Ace*-*1* mutation (N485I) associated with carbamate resistance in southern African *An. funestus* populations [28] coupled with the presence of the A296S-RDL mutation in the GABA receptor of *An. funestus* [20] are evidence that target-site resistance mechanism also play a role in insecticide resistance profiles recorded in this malaria vector.

In order to help malaria control programmes, to design evidence-based strategies to control *An. funestus* in Nigeria, and to manage potential existing resistance, this study aims to establish the insecticides susceptibility profile and investigate the molecular basis of resistance of this species population in Akaka Remo: a farm settlement in southwest Nigeria.

## Methods

### Ethical statement

No ethical permit was required for this study. However, there was a focus group discussion with the community and household heads where verbal consent was obtained for mosquito collections in the community after the study aims and objectives were explained.

### Study site and mosquito collection

#### Study site description

Akaka-Remo (6°57′N, 3°43′E) is a rural locality in Remo-North local government of Ogun state in the Southwest of Nigeria, a region of about 71.4 km from Lagos and about 215 km from Pahou in Benin where resistance has previously been reported. This locality is surrounded with a permanent medium-size slow moving stream, called Erititi stream, that leads to the popular river Ona in Ibadan (Oyo state) with vegetation such as bananas, vegetables, maize, shrubs, trees and crops bordering the water bodies at almost all the locations, which serves as suitable breeding sites for *An. funestus*. The inhabited area of Akaka-Remo is about 0.25 square kilometres and its habitants are mainly the Yorubas and a small community of the Eguns. The main commercial activity is agriculture, which has attracted the use of pesticides (insecticides and herbicides) in this locality. Houses here are mainly made of mud and very few are made of cement, and are constructed at an average of 5 m away from one other. Most houses have either detached/destroyed or no ceilings. The selection criteria described above were mainly entomological as the main target for this research was to characterise populations of malaria vectors in this part of Nigeria.

#### Mosquito collection

Adult female *Anopheles* mosquitoes resting indoor were collected in thirty rooms, with the use of electric aspirators and torches between 06.00 a.m. and 10.00 a.m. from October, 2014 end of rainy season to April, 2015 beginning of rainy season except in January, 2015 due to intense harmattan (a short period of a very dry and dusty wind observed between the end of November and early March in West Africa). The 30 rooms were selected in a way to cover the various micro-ecologies found at Akaka Remo. The room was defined as a demarcated area in the house where inhabitants do sleep. Blood-fed mosquitoes collected were kept into cups until fully gravid before being subjected to the forced-egg laying technique [30] at a temperature of 25–28 °C with a relative humidity of 80% in the insectary. Hatched eggs were pooled and reared together in a mineral water, which was replaced every two days to reduce mortality as resulting larvae were daily fed with Tetramin™ baby fish. During these periods, a good number of eggs were also sent via courier to the Liverpool School of Tropical Medicine (LSTM) for rearing into F_1_ and for subsequent experiments.

### Seasonal determination of mosquito densities per room

Mosquito density per room was estimated during four (4) annual climatic seasons: rainy season, transition from rainy to dry season, dry season and transition from dry to rainy season. The total number of *An. funestus* collected for each season were pooled and counted to estimate the seasonal number of *An. funestus* per room. The estimated density of *An*. *funestus* was now obtained by dividing the number of mosquitoes collected during each season by the number of rooms surveyed during that same season. This estimation of the density was done for the 4 surveyed seasons of the year.

### PCR-species identification

A total of 96 mosquito females that were morphologically identified as *An. funestus* group [31] and had oviposited, were identified to species level using the PCR cocktail for *An. funestus* group described by Koekemoer et al. [32] after the genomic DNA was extracted [33].

### Estimation of *Plasmodium* infection in wild caught (F_0_) *Anopheles funestus*

Ninety-three (93) F_0_ adult female *An. funestus* were analysed for *Plasmodium* infection using the TaqMan assay as previously described [34]. Briefly, a plate was run at one cycle of 95 °C for 10 min in the first segment and the second segment was 40 cycles at 92 °C for 15 s and 60 °C for 1 min. Two fluorophore-labelled specific TaqMan probes (Applied Biosystems, California, USA) were used: FAM to detect *Plasmodium falciparum,* while HEX was used to detect the combination of *Plasmodium ovale, Plasmodium vivax* and *Plasmodium malariae*. A negative control (water) and positive controls (known FAM and OVM) were also used. A nested PCR [35] was subsequently performed for the samples to validate the TaqMan analysis.

### Insecticide susceptibility tests

2–5 day old F_1_ adult female and male mosquitoes pooled from different F_0_ mosquitoes were used for this test according to the WHO [36]. 20–25 mosquitoes per tube with at least 4 replicates were exposed to insecticide-impregnated or control papers for 1 h before transferring into clean holding tube with 10% sugar solution where mortality was determined after 24 h post insecticide exposure [37]. Six insecticides belonging to the four classes of insecticides used for malaria vector control were tested: the pyrethroids permethrin (0.75%) and deltamethrin (0.05%), the organochlorines DDT (4%) and dieldrin (4%), the carbamate bendiocarb (0.1%) and the organophosphate malathion (5%).

### PBO synergist tests

Due to the level of resistance observed against DDT, dieldrin and permethrin and because of previous studies showing strong involvement of P450 genes in pyrethroids resistance as well as its potential contribution to DDT resistance, 2–5 days old F_1_ adult mosquitoes were pre-exposed to 4% piperonyl butoxide (PBO) paper for 1 h and immediately exposed to 0.75% permethrin, 4% DDT and 4% dieldrin for 1 h. Although, there is no previous data linking P450 families to dieldrin resistance in this mosquito population but with the high resistance observed in this study, it became necessary to assess the potential effect of oxidase in diedrin resistance. Mortalities were later assessed after exposure; synergized group was compared to the un-synergized group after 24 h post-exposure. This comparison was used to evaluate the potential role of cytochrome P450 genes in the observed resistance. Two controls were used during this experiment: control 1 was constituted of mosquitoes exposed to papers neither with insecticides nor with PBO while control 2 was constituted of mosquitoes exposed to papers treated with PBO only.

### Genotyping of resistance markers L119F-GSTe2 and A296S-RDL in females of *An. funestus* from Akaka-Remo

TaqMn assay [34] was used to genotype L119F-GSTe2 as a potential DDT resistance marker in Akaka-Remo, which was recently shown to confer DDT resistance in Benin [27] and also used to screen for A296S-RDL mutation known to confer dieldrin resistance [20]. F_0_ and F_1_ (alive and dead) samples were used for both genotyping. Two fluorophore-labelled specific TaqMan probes were used: FAM to detect the homozygous resistant genotype, HEX to detect the homozygous susceptible genotype while both FAM and HEX were used to detect the heterozygous genotype. A negative control (water) and positive controls (known FAM, HEX and both) were also used in a 10 µl volume that also contains the SensiMix (Applied Biosystems, California, USA). The endpoint fluorescence was evaluated using the Agilent MXPro software and the relationship between the frequency of the resistant alleles and the insecticides (DDT and Dieldrin) resistance phenotypes were assessed.

### Data analysis

Resistance status of mosquito classified as recommended by WHO [37] are as follows:

- Susceptible mosquito population = Mortality >98%.
- Suspected resistance in mosquito population = Mortality ranging from 90 to 98%.
- Resistant mosquito population = Mortality <90%.

Chi square (using R software) was used to test for significant difference in percentage mortalities between female and male mosquito populations used for WHO susceptibility test and the distribution of the genotype frequencies (F_1_) between the resistant and susceptible mosquito samples. Had2know online statistical software [38] was used to test for significant difference between the observed genotypic frequencies (F_0_) and to confirm if the observed genotypic frequencies are according to Hardy–Weinberg equilibrium. Excel was used to compute percentage mortalities and standard errors while VassarStats online statistical software [39] was used to generate odd and risk ratios, and confidence levels of the frequency data.

## Results

### Species identification

**Fig. 1:**
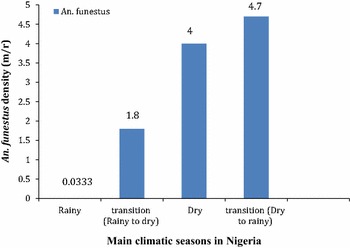
Seasonal density of *An. funestus* per room at Akaka-Remo. *m/r* mosquitoes per room

Molecular (PCR) analysis of ninety-six (96) morphologically identified female *An. funestus* sensu lato collected from Akaka-Remo between October, 2014 (late rainy season) and April, 2015 (early rainy season) revealed that they all belong to *An. funestus s.s. Anopheles funestus* is the most abundant mosquito species (84%; n = 315 from a total of 376 mosquitoes collected) amongst other mosquito species and much more abundant (92%; *An. funestus* = 315 and 8%; *An. gambiae* = 26) than *An. gambiae* when compared within the *Anopheles* group. Figure 1 shows the seasonal variation of *An. funestus s.s.* per room at Akaka-Remo. The seasonal density of *An. funestus* per room (m/r) out of thirty (30) rooms aspirated are as follows: 0.03 m/r for rainy season, 1.8 m/r during transition from rainy to dry, 4 m/r during dry and 4.7 m/r during the transition from dry to rainy season. Other mosquito species also collected during these periods include *An. gambiae* spp. (7%; n = 26), *Culex* spp. (6%; n = 21), *Mansonia* spp. (2%; n = 9) and *Aedes* spp. (1%; n = 5).

### Plasmodium infection rates

Seven, 7 (8%) out of ninety-three (93) wild-caught (F_0_) *An. funestus s.s.* analysed were positive for *Plasmodium* infection (Table 1). Six (7%) mosquitoes of which were infected with *P. falciparum,* while a mixed infection of *P. ovale, P. vivax* and *P. malariae* was found in only one mosquito (1%). The nested PCR analysis showed the presence of *P. falciparum* in 4 (4%) mosquitoes.

**Table 1:** *Plasmodium* infection rates of *An. funestus* from Akaka-Remo

Locality	Species Id	F_0_ tested	+ve *P. fal*	+ve *P. OVM*	+ve *P. fal* and *OVM*	Total infected with TaqMan (% infection)	Total infected with nested PCR (% infection)
Akaka-Remo	*An. funestus s.s.*	93	6	1	–	7 (8)	4 (4)

*fal* *falciparum, OVM* the combination of *ovale, vivax* and *malariae*

### WHO susceptibility tests

**Fig. 2:**
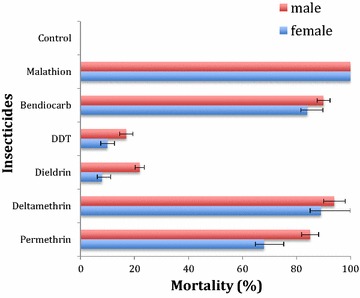
Insecticide resistance profiles of *An. funestus s.s*. from Akaka-Remo. *Error bars* represent standard error of the mean

A total of 96 F_0_ *An. funestus* oviposited out of the 315 samples collected on the field producing 1269 F_1_ adults (679 females and 590 males), which were all exposed to six different insecticides (Fig. 2). The highest level of resistance was recorded with organochlorines. Dieldrin exposure resulted into mortalities of 8% ± 3.24 (females) and 22% ± 1.73 (males). Likewise, DDT exposure produced mortalities of 10% ± 2.66 in females and 17% ± 2.45 in male populations. Resistance was also observed against both type I and II pyrethroids (without and with cyano group), with a mortality of 68% ± 5.64 in females (85% ± 3.15 for males) for permethrin (type I) and a mortality of 87% ± 10.96 (94% ± 3.98 for males) for deltamethrin (type II). In addition, bendiocarb (carbamate) resistance was also observed with mortalities of 84% ± 5.67 in females and 90% ± 2.36 for males. In contrast, a full susceptibility of 100% mortality was recorded in both females and males populations exposed to the organophosphate malathion. Overall, there was no significant difference (*χ* ^*2*^ = 7.73, *df* = 5, P = 0.172) in the percentage mortalities between the exposed females and males mosquitoes.

### Synergist tests with PBO

**Fig. 3:**
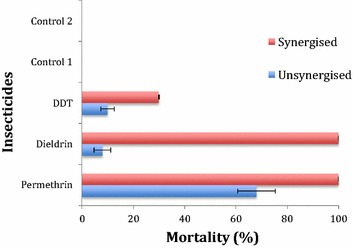
Insecticide resistance profiles of *An. funestus* after exposure to PBO. *Error bars* represent standard error of the mean; *Control 1* Mosquitoes that were neither exposed to PBO nor insecticides; *Control 2* Mosquitoes that were exposed to only PBO

There was a recovery of susceptibility to permethrin as mortality rose from 68 to 100% (n = 70) when permethrin was combined with the P450 inhibitor, PBO (Fig. 3). This suggests a likely significant role of cytochrome P450s in the pyrethroid resistance. Similarly, 100% mortality was recorded when PBO was combined with dieldrin. This unexpected recovery of susceptibility from 8 to 100% (n = 48) also implicates oxidases in dieldrin resistance. However, the combination of DDT with PBO only showed a slow increase in mortality from 10 to 30% (n = 45) suggesting a limited implication of P450 s in DDT resistance. No mortality was observed in the control mosquitoes exposed to control paper with no insecticide or only to PBO.

### Genotyping and allelic distribution of L119F-GSTe2 mutation in the *An. funestus* population from Akaka-Remo

**Fig. 4:**
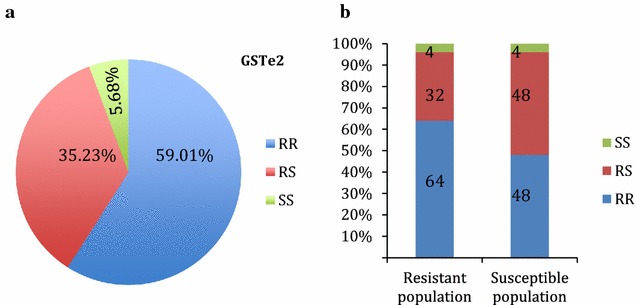
Screening of L119F-GSTe2 mutation (**a**) shows a high presence of RR and RS individuals and a low presence of SS in F_0_ females *An. funestus* from Akaka-remo. **b** F_1_ *An. funestus s.s*. from Akaka-Remo showing high presence of RR and RS and a relatively low presence of SS individuals in both the resistant (alive) and susceptible (dead) individuals post DDT exposure

The L119F-GSTe2 mutation was detected in 94% of the F_0_ mosquitoes (n = 88) that were genotyped (Fig. 4a). Over half (52) of the total mosquitoes analysed were homozygous resistant RR, 31 were heterozygous RS while just 5 were homozygous susceptible, SS with allelic frequencies of R = 77% and S = 23%. Similarly, when the F_1_ generations (25 resistant and 25 susceptible after bioassays with DDT) were screened for L119F-GSTe2 mutation, a genotypic frequency of 64% RR, 32% RS, and 4% SS and 48% RR, 48% RS and 4% SS were produced in the resistant and susceptible populations respectively. These resulted into allelic frequencies (119F) of 80% in the resistant and 72% in the susceptible populations (Fig. 4b). The observed genotypic frequency was shown to be at Hardy–Weinberg equilibrium (P = 0.8935). However, there was no significant difference (*χ* ^*2*^ = 1.37, *df* = 2, P = 0.5037) in the frequency of L119F-GSTe2 mutation between the susceptible and resistant samples and consequently the correlation was also not significant (OR = 1.56; P = 0.1859).

### Genotyping and allelic distribution of A296S-RDL mutation in *An. funestus* s.s. from Akaka-Remo

**Fig. 5:**
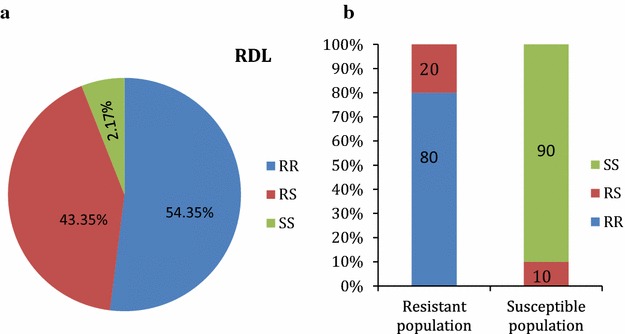
Genotype distribution of A296S-RDL mutation (**a**) showing a significant presence of RR and RS individuals in the F_0_ population of *An. funestus* from Akaka Remo (**b**) F_1_ *An. funestus s.s*. from Akaka-Remo post dieldrin exposure showing high presence of RR individuals in resistant (alive) and the absence of RR and low presence of RS in susceptible (dead) individuals

The A296S-RDL mutation was high in the F_0_ population (92 individuals genotyped) with homozygote RR claiming over half of the total mosquito analysed (50). Likewise, 40 samples were heterozygous RS and just 2 being homozygote susceptible SS (Fig. 5a). The high presence of A296S-RDL mutation in F_0_ mosquitoes correlates with the elevated phenotypic dieldrin resistance in this population (8 and 22% for females and males, respectively). But when F_1_ mosquitoes generated after bioassays with dieldrin (15 alive and 5 dead) were genotyped for A296S-RDL mutation, there was a high presence of the mutation in resistant population (genotypic frequency of 80% RR and 20% RS) with a relatively low presence in susceptible (genotypic frequency of 10% RS) sample. These produced allelic frequencies (296S) of 90% and 10% in the resistant and susceptible populations respectively (Fig. 5b). The observed genotypic frequency was also shown to be at Hardy–Weinberg equilibrium (P = 0.0617). There was a significant difference (*χ* ^*2*^ = 16, *df* = 2, P = 0.00034) in the frequency of A296S-RDL mutation in the resistant population compared to the susceptible sample and consequently correlation was also significant (OR = 81; P < 0.0001).

## Discussion

### Role of *An. funestus* in malaria transmission at Akaka-Remo

*Anopheles funestus* is the most abundant mosquito species (84%) recorded at Akaka-Remo during the sampling period. Other mosquito species identified include *An. gambiae s.l.*, *Culex* spp, *Aedes* spp. and *Mansonia* spp.; these represent 26% of sampled mosquitoes. Among the malaria vectors identified in this locality, *An. funestus* density was over 10 times higher that *An. gambiae* (92 and 8% respectively). This present report is in contrast with the report of Oyewole et al. [4] in 2005, where *An. funestus* collected, n = 85 was nowhere near that of *An. gambiae,* n = 500. This study supports previous reports in Ogun [4], Lagos [6], Oyo and Kwara states [5] that *An. funestus s.s*. is a major malaria vector in Nigeria with a confirmation of 8% *Plasmodium* infection in this mosquito species. It therefore emphasizes the importance of this vector and the threat it could pose to malaria transmission in the Southwest of Nigeria. The 8% infection rate observed in Akaka-Remo is similar to high levels of infection rates recorded previously for *An. funestus* across the continent such as the 20% [24] and 50% [40] observed in Burkina Faso, the 13.6% [41] and 18% [26] observed in Benin and 12.5% in Ghana [42]. Although, some of the variations between these rates could be down to the differences in the methods used (TaqMan, ELISA and Nested-PCR) and the consistent high levels support a high vectorial capacity of *An. funestus* exhibits across the continent.

Another common member of the *An. funestus* group, *Anopheles rivulorum* that was previously identified both indoor and outdoor at Akaka-Remo [4] was absent in this study potentially due to a change in resting preference of this species although more entomological studies are needed to explain this change. Previous reports on the other known malaria vector, *An. gambiae* in Nigeria have shown that infection rates range mostly between 2 and 8.1% [4–6, 43]. This research has shown the in-houses abundant presence of *An. funestus* at Akaka-Remo compared to *An. gambiae* during all climatic seasons. It has also revealed the consistent level of mosquito infections with *Plasmodium* species (8% infected mosquitoes) in this locality hence, highlighting the fact that *An. funestus* plays a significant role in malaria transmission in this community in southwest Nigeria.

### Multiple insecticide resistance at Akaka-Remo

This study reports that *An. funestus s.s*. in Akaka-Remo has developed resistance to most common public health insecticides. Results from this research highlight the presence of multiple insecticide resistance in this malaria vector. Most studies on insecticide resistance in Nigeria have focused on *An. gambiae* with less interest on *An. funestus* as this vector was essentially thought to be susceptible to pyrethroids, the main insecticide used for malaria vector control. This study has shown that this *An. funestus* population has not only become resistant to pyrethroids but to a wide range of public health insecticides from other families. The multi-insecticide resistance pattern observed in Akaka-Remo is similar to what was previously reported in Benin [22, 26].

The level of DDT resistance observed from Akaka Remo is as high as the case in Benin. This present report is higher than what was observed in Uganda and Kenya (40–42% mortality) [12], and in Malawi (69.9% mortality) [17] among others. The high DDT resistance recorded in Benin (Pahou) and now in Nigeria (Akaka Remo), both in West Africa compare to a relatively lower resistance profiles in the East and the Southern Africa might be as a result of different genetic make-up of this species between regions of Africa. Resistance recorded with dieldrin is not only the highest of all the six insecticides tested but also the highest recorded in Africa until now. Resistance level (8% mortality) is higher than Burkina-Faso (30% mortality) [20] and Benin (93.3% mortality) populations [22]. In Southern Africa, dieldrin susceptibility has been frequently observed until a recent report of resistance in Malawi (83.9% mortality) [17]. Organochlorines resistance recorded in this vector at Akaka-Remo coupled with reports of DDT resistance in *An*. *gambiae* [10] will obviously further disapproves the re-introduction of this insecticide family as an alternative to pyrethroids for mosquito control in Nigeria. DDT and dieldrin resistance recorded in this study could be associated with the residual effect of the long historical usage of this insecticide family (organochlorine) in agriculture when this sector was a key source of income in Nigeria [44]. The oil boom in the 1970s latter shifted the national attention from agriculture to the oil and gas sectors. This economic sector (oil production) was more lucrative than agriculture and became the main asset for Nigeria [45]. One could also argue that resistance may be due to the poor attitudes and/or ignorance of farmers towards observing good farming practices when using this insecticide family to control pests [46, 47]. During mosquito collections, it was observed that some of the villagers even make use of these agrochemicals to control insects at home. Such ignorance could add-on to the high DDT and dieldrin resistance in this study area. It is therefore important to determine the extent to which *An. funestus* has developed resistance to this insecticide family by investigating its spread across different geographical regions in Nigeria.

In Nigeria, agrochemicals use are approved by the National Agency for Food and Drug Administration and Control, NAFDAC [48]. It is possible that the misuse and/or over use of these chemicals by farmers could be fundamental for the multi-resistance selection in this locality. The indiscriminate use of agro-chemicals by farmers could have also generated high chemical residues and other environmental pollutants that are washed into the water bodies (mosquito breeding sites) generating several xenobiotics that exercise a resistance selection in mosquitoes at larval stage [49–52]. Similarly, spilled petroleum products found in several mosquito breeding sites in the south–western Nigeria [53] might have also contributed to resistance selection in *An. funestus* from Akaka Remo through cross resistance mechanisms. Both environmental factors (generation of chemical pesticides and spillage of petroleum products) are common in Nigeria and can certainly contribute to the local selection of the observed insecticide resistance profiles. Further assessments are still needed to clearly map out the actual factor(s) contributing to the multi-insecticide resistance in *An. funestus* population from Akaka-Remo.

Pyrethroid resistance on the other hand is high but not as the previous two insecticides (DDT and dieldrin). Susceptibility test with pyrethroids is important because the country depends on this insecticide family for malaria vector control [2]. Mortalities recorded with permethrin (68%) and deltamethrin (87%) are similar to what was reported in Pahou (permethrin = 66.7% and deltamethrin = 88.60%) [22] but higher than Kpome (permethrin = 13.03% and deltamethrin = 46.49) [26]. These reports show that pyrethroids resistance is increasing in the West Africa population of *An. funestus*. The pattern of pyrethroids resistance in Western Africa is different from East [12] and Southern Africa [14], where resistance to deltamethrin is higher than permethrin. Pyrethroids resistance recorded in *An. funestus* from Akaka-Remo is a great concern for malaria control programs and there is a risk that this mosquito species would have developed resistance to pyrethroids in different regions of Nigeria due to the current heavy use of this class of insecticide both in agriculture and public health all over the country. If this happens, it will constitute more ordeals for future malaria vector control interventions through the use of pyrethroid-based insecticides for both ITNs and IRS. These findings therefore suggest further studies to determine the extent of pyrethroid resistance in *An. funestus* populations in Nigeria.

Bendiocarb resistance (84% mortality) in this species population is also a concern because carbamate-based insecticide interventions were recently introduced as an alternative to pyrethroid-based in West Africa. Bendiocarb resistance was also reported in Pahou (65% mortality) in 2011. Bendiocarb resistance is higher in Southern Africa: Zambia, Zimbabwe and Mozambique [16, 25, 54] but lower in East Africa [12] compared to West Africa. The current level of bendiocarb resistance raises an alarm as it might affect the success of the recently introduced bendiocarb-based IRS in West Africa. Hence, it will be important to have more information on the potential spread of resistance across different regions and some underlying factors that might be responsible. This information will guide the malaria control programs to improve its subsequent release of bendiocarb-based IRS. The organophosphate malathion is really proving to be the most reliable insecticide considering similar records of full susceptibility in *An. funestus* population all over Africa. This insecticide could, therefore, be used as an alternative insecticide to manage resistance.

### Underlying mechanisms of the observed multiple insecticide resistance patterns at Akaka-Remo

The proven absence of the *kdr* mutation in *An. funestus* populations from Benin (a neighbouring country) [22] and other regions in Africa [12] coupled with the high mortality observed when permethrin was combined with PBO in this study support the fact that pyrethroid resistance is still driven by metabolic resistance mechanisms. The use of the synergist PBO revealed the role of oxidase, notably cytochrome P450s, in pyrethroid resistance of this mosquito population similar to what have been reported so far in Africa [12, 26]. However, the glutathione S-transferase gene, GSTe2 was shown to play a higher role in DDT resistance in Benin through over-transcription and also the selection of the resistant allele L119F [27]. The high frequency of L119F in Akaka-Remo (77% in F_0_) associated with the high DDT resistance level support a significant role for the L119F-GSTe2 mutation in the DDT resistance in this *An. funestus* population. However, this high frequency of L119F in this location is probably the reason why a lack of correlation was observed when comparing resistant and susceptible samples as observed regularly for *kdr* mutations such as L1014F in *An. gambiae* in situation when the 1014F resistant allele is nearly fixed [55]. The L119F-GSTe2 mutation has also been detected in other DDT resistant populations such as Ghana (44.2%) and Burkina-Faso (25%) in West Africa, and Cameroon (48.2%) in Central Africa [20, 23] as well as Uganda (20.4%) and Kenya (7.8%) in East Africa [12]. In Southern Africa (Malawi) however, L119F allele is absent despite the recent detection of DDT resistance in this region suggesting a different DDT resistance mechanism in this *An. funestus* population [17].

Dieldrin resistance on the other hand showed a strong association with oxidase. This is unexpected because dieldrin resistance has always been linked with target-site insensitivity [20]. Also, mutation detected on *GABA* receptor (A296S-RDL) gene in the parent and first filial generation of this *An. funestus* population indicates that this mosquito species adopts more than one mechanism for dieldrin resistance. More screening of dieldrin susceptibility should be done and further studies should be conducted to determine the geographical distribution of dieldrin resistance in *An. funestus* from Nigeria.

## Conclusion

This study reports the presence of multiple insecticide resistance in *An. funestus* population from Akaka-Remo in the southwestern Nigeria. Molecular analysis conducted in the course of this research have revealed that this *An. funestus* population have developed multiple resistance mechanisms to withstand lethal doses of insecticides used in public health. The consistent implication of *An. funestus* in malaria transmission at Akaka-Remo was also established in this study. Nevertheless, further studies are needed to determine the spread of insecticide resistance and to conduct more investigations on underlying mechanisms of insecticides resistance for improved malaria control strategies in Nigeria.

## Abbreviations

DDT: dichlorodiphenyltrichloroethane
NAFDAC: National Agency for Food and Drug Administration and Control
m/r: mosquito per room
spp: species
LSTM: Liverpool School of Tropical Medicine
PBO: piperonyl butoxide
RDL: resistance to dieldrin
PCR: polymerase chain reaction
OR: odd ratio
WHO: World Health Organization
